# Euglycemic diabetic ketoacidosis induced by sodium-glucose cotransporter 2 inhibitor in the setting of prolonged fasting: a case report

**DOI:** 10.1186/s13256-022-03347-1

**Published:** 2022-03-29

**Authors:** Abrar Alkatheeri, Eiman Alseddeeqi

**Affiliations:** 1 Department of Medical Education, Medical Affairs, Sheikh Khalifa Medical City, P.O. Box 51900, Abu Dhabi, United Arab Emirates; 2 Division of Endocrinology, Sheikh Khalifa Medical City, P.O. Box 51900, Abu Dhabi, United Arab Emirates

**Keywords:** Euglycemic diabetic ketoacidosis, EDKA, DKA, Sodium-glucose cotransporter 2 inhibitor, Dapagliflozin, Prolonged fasting, Case report

## Abstract

**Background:**

We describe a case report of a patient with type 2 diabetes on sodium-glucose cotransporter 2 inhibitor and metformin therapy fasting for Ramadan (a holy month observed in the Islamic nation) diagnosed with euglycemic diabetic ketoacidosis.

**Case presentation:**

The patient was a 51-year-old Moroccan male with history of type 2 diabetes mellitus on dapagliflozin and metformin. He presented with abdominal pain, vomiting, loss of appetite, and shortness of breath. He observed Ramadan month by fasting an average of 14 hours daily for 30 days. The patient was admitted with severe metabolic acidosis with a high anion gap and positive ketonuria in the setting of serum glucose of 13.5 mmol/L (243 mg/dL).

The patient was rehydrated and started on insulin infusion according to the diabetic ketoacidosis protocol following the diagnosis of euglycemic diabetic ketoacidosis.

**Conclusion:**

Dapagliflozin is associated with euglycemic diabetic ketoacidosis in the setting of prolonged fasting. Counseling and possible medication adjustment should be added to clinical practice in those planning to decrease caloric intake through dedicated fasting including Ramadan or weight-loss-directed behavioral modifications, especially if taking sodium-glucose cotransporter 2 inhibitors.

## Background

Diabetic ketoacidosis (DKA) is defined by certain diagnostic criteria, including blood glucose level of more than 13.9 mmol/L (250 mg/dL), acidosis (arterial pH < 7.3 and serum bicarbonate < 15 mEq/L), and ketonemia [1]. Euglycemic diabetic ketoacidosis (EDKA) is defined as follows: blood glucose level below 13.9 mmol/L (250 mg/dL), acidosis, and development of ketonemia [2, 3].

The first case of EDKA was described by Munro in 1973, whereby he studied 211 cases of DKA, 11 of which had blood glucose levels below 16.7 mmol/L (300 mg/dL) [4]. This was classically considered a rare incident, but it is likely underreported [2]. The etiology of EDKA is unknown, but there are many stressors and conditions that can precipitate an episode in either a diabetic or healthy patient [5, 6]. Such stressors include fasting, anorexia, gastroparesis, being on a ketogenic diet, and higher alcohol consumption [3, 7]. Furthermore, conditions such as pregnancy, cirrhosis, pancreatitis, insulin pump use, sepsis, cardiovascular events, and recent major surgery also play a similar role [3, 8].

The benefits of SGLT2 inhibitors have been extensively studied, including adequate glycemic control, weight loss, and more importantly, nephrological and cardiovascular protection [10–12]. Alternatively, life-threatening side-effects related to this class of medication, such as diabetic ketoacidosis and EDKA, have also been documented in literature [13]. Therefore, a warning is included in the drug label for SGLT2 inhibitors [9]. These medications, including canagliflozin, dapagliflozin, and empagliflozin, increase the risk of inducing EDKA [3, 15, 16]. Triggers inducing EDKA predispose the body to a carbohydrate deficit, which will result in lipid catabolism as a source of energy via the anaerobic pathway. Fatty acids are then produced by lipolysis, and high levels of fatty acids leads to ketone formation [3, 6]. Here, we present the first case of EDKA associated with 14 hours of fasting daily for 30 days while being on dapagliflozin.

The ritual of fasting in the Islamic nation is most significant in Ramadan. Ramadan is the month of fasting, which is a deed practiced by many Muslims. Muslims fast for thirty consecutive days from sunrise to sunset, and therefore, hours of fasting change from one country to another but generally range from 10 hours to 21 hours. Fasting during this time includes all forms of food or liquids, including water. At sunset, Muslims break their fast when they start consuming meals and drinks. Ramadan is followed by a huge population in the Islamic world, leading to the importance of addressing clinical risks associated with prolonged fasting in patients with diabetes, especially when treated with SGLT2 inhibitors [17]. Here we present the first case of EDKA associated with Ramadan fasting that included a 14-hour daily fast sustained over a 30-day period while being on dapagliflozin.

## Case presentation

A 51-year-old Moroccan male with past medical history of type 2 diabetes mellitus presented to the emergency department for evaluation of his acute symptoms. He complained of epigastric pain and fatigue for 3 days. He was further reported to have bouts of nonbloody, nonbilious vomiting associated with poor oral intake. He denied any chest pain, fever, or cough. He had no change in bowel habits. He reported no contact with a sick person or recent travel history. His family and psychosocial histories were unremarkable. Upon documenting his medication history, the patient was on metformin regularly for 10 years for his diabetes. Dapagliflozin 5 mg was added to metformin 1000 mg daily 12 weeks prior to presentation. The symptoms started the day following completion of Ramadan fasting over a 30-day period. Fasting from food and drinks lasted daily for approximately 14 hours daily. He broke his fast with a three-course meal, starting with a light meal which was mainly based on fruits and fluids, a second course of protein-based items along with vegetables and carbohydrate-containing items. Finally, the meal was concluded with carbohydrate-based items. Drinks were consumed throughout the fast-breaking period.

Vital signs in the emergency department were documented as follows: Kussmaul’s pattern of breathing, with a respiratory rate of 22 breaths/min and a pulse rate of 112 bpm. The patient was otherwise alert and oriented. His chest and abdominal examination were unremarkable. DKA workup, blood sugar level, venous blood gas, and urine analysis were carried out; the results are presented in Table 1.

**Table 1:** Metabolic panel

Labs	Reference range	ED	Day 1	Day 2
Random blood glucose	3.9–6.1 mmol/L (70.2–109.8 mg/dL)	13.5 (243)	6.4 115.2	9.2 165
Sodium	136–145 mmol/L	143	142	136
Potassium	3.4–5.1 mmol/L	4.4	3.7	3.6
Chloride	98–107 mmol/L	109	110	109
Venous blood gas				
pH		6.92	7.2	7.3
PaCO_2_	35.0–45.0 mmHg	20.4	24.7	41.1
HCO_3_	35.0–40.0 mmol/L	4	11	25
Anion gap	8–16 mEq/L	30	21	2
Serum lactate	0.5–2.2 mmol/L	2.50	0.7	0.6

Initial blood workup revealed a random blood glucose of 13.5 mmol/l (243 mg/dL). The venous blood gas results showed severe metabolic acidosis with a high anion gap, pH of 6.9 and PCO_2_ of 20.4 mmHg with HCO_3_ of 4 mmol/L. The calculated anion gap was 30 mEq/L. Urine was positive for ketones and glucose. Based on the above presentation, euglycemic diabetic ketoacidosis was diagnosed. The patient was resuscitated with 4 L of normal saline intravenously, and insulin at a dose of 1 unit/kg was initiated.

The cause of ketoacidosis was investigated to rule out other precipitants for acidosis. Complete blood work-up showed a mild increase in white blood cells at 16.5 × 10^9^/L (normal range of 4.5–11.0 × 10^9^/L) with a high neutrophil count of 14.56 × 10^9^/L (normal range of 1.8–7.70 × 10^9^/L). Inflammatory markers, such as C-reactive protein, were within the normal range. Furthermore, a 12-lead electrocardiogram showed a sinus rhythm with minimal nonspecific changes in the inferior leads, which could be related to increased respiration. However, cardiac enzymes were within normal range. The patient did not report any respiratory symptoms; therefore, a chest X-ray was not indicated.

He was admitted to a regular medical ward and was kept on insulin and sodium bicarbonate infusions. Metformin and dapagliflozin were not resumed. The patient’s condition improved and as did his metabolic parameters (Table 1). The blood gas normalized, and the anion gap closed. His capillary blood glucose readings were optimal for the inpatient setting. The patient’s insulin infusion was bridged to subcutaneous insulin. He was discharged on insulin glargine 10 units at bedtime and aspart insulin 5 units before meals.

## Discussion

We report a case of euglycemic diabetic ketoacidosis triggered by prolonged fasting. To the best of our knowledge, this is the first case report of EDKA with SGLT2 inhibitor use induced by decreased caloric intake due to Ramadan fasting. It is well known that prolonged fasting (as early as 12–14 hours of fasting) will result in ketone formation. Ketone formation normally augments insulin secretion, which can decrease the rate of ketone generation; however, in diabetic patients or those who use SGLT2 inhibitors, this mechanism is affected [3, 6].

Other stressors have been briefly mentioned in literature and even as case reports, but in this case, the severity of acidosis was remarkable especially in SGLT2 inhibitor-induced EDKA.

It was challenging to arrive at a diagnosis on presentation as the patient’s symptoms were generalized and could fit many differentials. However, the Kussmaul pattern of breathing and tachypnea along with history of diabetes mellitus prompted doctors to check venous blood gas to rule out acidosis. EDKA could result from multiple precipitants, but decreased oral intake is the most cited stressor for precipitating EDKA with or without SGLT2 inhibitor use [2, 3, 5, 6]. In addition, studies have shown that SGLT2 inhibitors, in general, increase the risk of DKA by sevenfold [16]. According to a report generated by the Food and Drug Administration (FDA), seventy-three cases of ketoacidosis requiring hospitalization were reported from March 2013 to May 2015 in patients with type 1 or type 2 diabetes mellitus treated with SGLT2 inhibitors; however, out of 73 cases, 40 had an average blood glucose levels of 11.7 mmol/L (211 mg/dL).

Being on a carbohydrate-deficient diet leads to a hypoglycemic effect that will result in a shift from glucose to lipid utilization for energy production. This mechanism will also increase glucagon levels and decrease insulin stimulation, promoting ketogenesis [18, 19]. The exact mechanism by which SGLT2 inhibitors precipitate the development of EDKA is not yet clear. However, the proposed pathophysiological mechanism is that SGLT2 blockers decrease blood glucose levels by increasing glycosuria and decreasing endogenous insulin secretion. In addition, they directly stimulate α-cells to produce more glucagon in response to hypoglycemia and decrease insulin secretion. As a result of this effect, more glucagon is produced, which promotes hepatic ketogenesis. Another effect of SGLT2 inhibitors is an increase in the reabsorption of acetoacetate in the renal tubules, which contributes to the increase in ketone levels in the blood [16, 20, 21].

In 2021, Tan reported three cases of EDKA in diabetic patients on SGLT2 inhibitors that were induced by a recent decrease in caloric intake [13]. In contrast, our patient fasted for 14 hours daily but then broke his fast at sunset as part of observing Ramadan while being on dapagliflozin.

Possible precipitants mentioned above were ruled out upon our patient’s presentation, hence we presumed the cause of his ketoacidosis was fasting in Ramadan while being on dapagliflozin.

In 2017, a literature review described the effect of fasting for Ramadan on insulin-dependent diabetic patients. All studies concluded that there was no change but rather some improvement in glycemic control during fasting for Ramadan. Furthermore, the incidence of hyperglycemia and diabetes ketoacidosis was insignificant [22].

Similarly, a low-carbohydrate diet and an increase in fat in the diet can theoretically induce ketoacidosis [23]. There have been case reports illustrating the effect of a low-calorie diet consumed by diabetic patients while on SGLT2 inhibitors, leading to EDKA [5, 24]. In contrast, in our case, the patient had been fasting long enough where there was no intake of food and drinks for long hours. This denotes an association between fasting and the development of EDKA while on a SGLT2 inhibitor [24].

Typically, the treatment of SGLT2 inhibitor-associated EDKA is similar to the conventional DKA treatment protocol, with an emphasis on closer monitoring of capillary blood glucose levels. For this reason, our patient was fluid resuscitated and started on insulin infusion, and electrolyte disturbances were corrected [25]. Universally, as a preventative measure, it is advised to stop SGLT2 inhibitors in the setting of acute illness to decrease the risk of EDKA [26]. In clinical practice, the Australian and New Zealand Colleges of Anesthetists advocate that SGLT2 inhibitors be stopped 3 days prior to surgery or in other physically stressful situations [27].

The practice in our institution in preparation for Ramadan fasting is to educate patients on switching the time of medication intake and instruct them to take it once they break their fast. Moreover, we advise them to increase fluid intake to keep hydrated. It is of crucial importance to educate patients if they undergo any dietary changes, such as carbohydrate restriction or being on a ketogenic diet [24].

As a recommendation, patients on SGLT2 inhibitors are advised to keep themselves hydrated, have a balanced meal, and consider stopping the medication during prolonged periods of fasting and/or in case of illness [28]. A discussion between the care provider and patient should occur before initiating a SGLT2 inhibitor. Counseling should include mentioning adverse events, especially in the setting of prolonged fast, to avoid pronounced ketone formation and acidemia. This case highlights the importance of counseling, especially in patients using SGLT2 inhibitors.

## Conclusion

SGLT2 inhibitors can increase the risk of EDKA. More cases are now being reported as these medications are used in abundance in clinical practice. However, this is the first case report describing EDKA induced by prolonged fasting due to Ramadan while on a SGLT2 inhibitor. Physicians should have a high level of suspicion for EDKA if the patient is on a SGLT2 inhibitor. EDKA counseling and possible medication adjustment should be added to clinical practice in those on SGLT2 inhibitors who are decreasing caloric intake through weight-loss-directed behavioral modifications or dedicated fasting such as during the month of Ramadan.

## Data Availability

Data sharing was not applicable to this article as no datasets were generated or analyzed during the current study.

## Abbreviations

SGLT2: Sodium-glucose cotransporter 2
DKA: Diabetes ketoacidosis
EDKA: Euglycemic diabetic ketoacidosis

## References

[1] Kitabchi AE, Umpierrez GE, Murphy MB, Kreisberg RA. Hyperglycemic crises in adult patients with diabetes: a consensus statement from the American Diabetes Association. Diabetes Care. 2006;29(12):2739–48. 10.2337/dc06-9916. 17130218

[2] Peters AL, Buschur EO, Buse JB, Cohan P, Diner JC, Hirsch IB. Euglycemic diabetic ketoacidosis: a potential complication of treatment with sodium-glucose cotransporter 2 inhibition. Diabetes Care. 2015;38(9):1687–93. 10.2337/dc15-0843. PMC4542270 26078479

[3] Plewa MC, Bryant M, King-Thiele R. Euglycemic Diabetic Ketoacidosis. [Updated 2021 Jun 4]. In: StatPearls [Internet]. Treasure Island (FL): StatPearls Publishing; 2021. Available from: https://www.ncbi.nlm.nih.gov/books/NBK554570/. 32119457

[4] Munro JF, Campbell IW, McCuish AC, Duncan LJ. Euglycaemic diabetic ketoacidosis. Br Med J. 1973;2(5866):578–80. 10.1136/bmj.2.5866.578 PMC1592207 4197425

[5] Larroumet A, Camoin M, Foussard N, Alexandre L, Mesli S, Redonnet I, Baillet-Blanco L, Rigalleau V, Mohammedi K. Euglycemic ketoacidosis induced by therapeutic fasting in a non-diabetic patient. Nutrition. 2020;72:110668. 10.1016/j.nut.2019.110668. 31982728

[6] Joseph F, Anderson L, Goenka N, Vora J. Starvation-induced true diabetic euglycemic ketoacidosis in severe depression. J Gen Intern Med. 2009;24(1):129–31. 10.1007/s11606-008-0829-0. PMC2607495 18975036

[7] Legaspi R, Narciso P. Euglycemic diabetic ketoacidosis due to gastroparesis, a local experience. J Ark Med Soc. 2015;112(5):62–3. 26390536

[8] Guo RX, Yang LZ, Li LX, Zhao XP. Diabetic ketoacidosis in pregnancy tends to occur at lower blood glucose levels: case-control study and a case report of euglycemic diabetic ketoacidosis in pregnancy. J Obstet Gynaecol Res. 2008;34(3):324–30. 10.1111/j.1447-0756.2008.00720.x. 18588610

[9] *FDA revises labels of SGLT2 inhibitors for diabetes to include warnings about too much acid in the blood and serious urinary tract infections*. (2015, May 15). U.S Food and Drug Administration. Retrieved October 1, 2022, from https://www.fda.gov/drugs/drug-safety-and-availability/fda-revises-labels-sglt2-inhibitors-diabetes-include-warnings-about-too-much-acid-blood-and-serious.

[10] Bashier A, Khalifa AA, Rashid F, Abdelgadir EI, Al Qaysi AA, Ali R, Eltinay A, Nafach J, Alsayyah F, Alawadi F. Efficacy and safety of SGLT2 inhibitors in reducing glycated hemoglobin and weight in emirati patients with type 2 diabetes. J Clin Med Res. 2017;9(6):499–507. 10.14740/jocmr2976w. PMC5412523 28496550

[11] Sarafidis P, Ferro CJ, Morales E, et al. SGLT-2 inhibitors and GLP-1 receptor agonists for nephroprotection and cardioprotection in patients with diabetes mellitus and chronic kidney disease. A consensus statement by the EURECA-m and the DIABESITY working groups of the ERA-EDTA. Nephrol Dial Transplant. 2019;34(2):208–30. 10.1093/ndt/gfy407 30753708

[12] Zinman B, Wanner C, Lachin JM, Fitchett D, Bluhmki E, Hantel S, Mattheus M, Devins T, Johansen OE, Woerle HJ, Broedl UC, Inzucchi SE, EMPA-REG OUTCOME Investigators. Empagliflozin, cardiovascular outcomes, and mortality in type 2 diabetes. N Engl J Med. 2015;373(22):2117–28. 10.1056/NEJMoa1504720. 26378978

[13] Peters AL, Buschur EO, Buse JB, Cohan P, Diner JC, Hirsch IB. Euglycemic diabetic ketoacidosis: a potential complication of treatment with sodium-glucose cotransporter 2 inhibition. Diabetes Care. 2015;38:1687–93. 10.2337/dc15-0843 PMC4542270 26078479

[14] Tan KT. Three cases of euglycaemic diabetic ketoacidosis related to the use of sodium-glucoseco-transporter-2 inhibitors and calorie restriction. Singapore Med J. 2021;62(3):153–4. 10.11622/smedj.2021029 PMC8027152 33846756

[15] Rosenstock J, Ferrannini E. Euglycemic diabetic ketoacidosis: a predictable, detectable, and preventable safety concern with SGLT2 inhibitors. Diabetes Care. 2015;38(9):1638–42. 10.2337/dc15-1380. 26294774

[16] Blau JE, Tella SH, Taylor SI, Rother KI. Ketoacidosis associated with SGLT2 inhibitor treatment: analysis of FAERS data. Diabetes Meta Res Rev. 2017;33:e2924. 10.1002/dmrr.2924 PMC5950709 28736981

[17] Rouhani MH, Azadbakht L. Is Ramadan fasting related to health outcomes? A review on the related evidence? J Res Med Sci. 2014;19:987–92. PMC4274578 25538785

[18] Mendelsohn RA, Taveras AN, Mazer BA, Clayton LM. Euglycemic diabetic ketoacidosis precipitated by SGLT-2 inhibitor use, pericarditis, and fasting: a case report. Clin Pract Cases Emerg Med. 2020;4(3):389–92. 10.5811/cpcem.2020.4.46056. PMC7434240 32926693

[19] Nyenwe EA, Kitabchi AE. The evolution of diabetic ketoacidosis: an update of its etiology, pathogenesis and management. Metabolism. 2016;65(4):507–21. 10.1016/j.metabol.2015.12.007. 26975543

[20] Ogawa W, Sakaguchi K. Euglycemic diabetic ketoacidosis induced by SGLT2 inhibitors: possible mechanism and contributing factors. J Diabetes Investig. 2016;7:135–8. 10.1111/jdi.12401 PMC4773669 27042263

[21] Ferrannini E, Baldi S, Frascerra S, Astiarraga B, Heise T, Bizzotto R, Mari A, Pieber TR, Muscelli E. Shift to fatty substrate utilization in response to sodium-glucose cotransporter 2 inhibition in subjects without diabetes and patients with type 2 diabetes. Diabetes. 2016;65(5):1190–5. 10.2337/db15-1356. 26861783

[22] Alabbood MH, Ho KW, Simons MR. The effect of Ramadan fasting on glycaemic control in insulin dependent diabetic patients: a literature review. Diabetes Metab Syndrome Clin Res Rev. 2017;11(1):83–7. 10.1016/j.dsx.2016.06.028. 27402028

[23] Pankaj S, William L. Ketoacidosis during a low-carbohydrate diet. N Engl J Med. 2006;354(1):97–8. 10.1056/NEJMc052709. 16394313

[24] Tsutsui E, Hoshina Y, Homma H. Sodium-glucose cotransporter-2 inhibitor-induced euglycemic diabetic ketoacidosis followed by excessively low carbohydrate diet. Cureus. 2021;13(7): e16085. 10.7759/cureus.16085. PMC8325382 34345561

[25] Fayfman M, Pasquel FJ, Umpierrez GE. Management of hyperglycemic crises: diabetic ketoacidosis and hyperglycemic hyperosmolar state. Med Clin North Am. 2017;101(3):587–606. 10.1016/j.mcna.2016.12.011. PMC6535398 28372715

[26] UK Medicines and Healthcare Products Regulatory Agency SGLT2 inhibitors:updated advice on the risk of diabetic ketoacidosis. Available at: https://www.gov.uk/drug-safety-update/sglt2-inhibitors-updated-advice-on-the-risk-of-diabetic-ketoacidosis. Accessed November 7, 2021.

[27] Australian and New Zealand College of Anaesthetists Alert: Severe euglycaemic ketoacidosis with SGLT2 inhibitor use in the perioperative period. Available at: https://www.anzcagetattachment.edu.au//d16e5295-41da-44a2-a21b-3bce2e0e10ae/Periprocedural-Diabetic-Ketoacidosis-(DKA)-with-SGLT2-Inhibitor-Use. Accessed Novemeber 11, 2021.

[28] Committee on the Proper Use of SGLT2 Inhibitors. Recommendations on the proper use of SGLT2 inhibitors. J Diabetes Investig. 2020;11(1):257–61. 10.1111/jdi.13160. PMC6944824 31729165

